# Impact of IFN-Free and IFN-Based Treatment on Blood Myeloid Dendritic Cell, Monocyte, Slan-DC, and Activated T Lymphocyte Dynamics during HCV Infection

**DOI:** 10.1155/2020/2781350

**Published:** 2020-03-16

**Authors:** Serena Vita, Paola Zuccalà, Stefano Savinelli, Claudia Mascia, Raffaella Rossi, Francesco Schiavone, Raffaella Marocco, Tiziana Tieghi, Marco Iannetta, Parni Nijhawan, Maria Antonella Zingaropoli, Gabriella d'Ettore, Vincenzo Vullo, Claudio Maria Mastroianni, Miriam Lichtner

**Affiliations:** ^1^Department of Public Health and Infectious Disease, Sapienza University of Rome, Italy; ^2^S.M. Goretti Hospital, Latina, Italy; ^3^Department of Molecular Medicine, Sapienza University of Rome, Italy

## Abstract

Chronic hepatitis C virus infection leads to the activation of innate immunity, a key component in HCV fibrosis. In the past, the use of IFN-based treatment regimens did not permit an adequate evaluation of the impact of HCV clearance on immune cells, because of their antiviral and immunomodulatory properties. The recent development of direct-acting antiviral (DAA) therapy, which is associated with high rates of sustained virological response, enables a more accurate analysis of the immunological modifications following HCV eradication. We studied the dynamics of blood myeloid dendritic cells, monocytes, slan-DCs, and T lymphocytes during IFN-free and IFN-based regimens in hepatitis C virus infection.

## 1. Introduction

Worldwide, an estimated 71 million people are chronically infected with hepatitis C virus (HCV) [1].

Chronic HCV infection is characterized by an aberrant inflammatory response that causes HCV-mediated liver damage, leading to progressive fibrosis, potentially resulting in cirrhosis, liver failure, and hepatocarcinoma (HCC) [2, 3].

HCV can counteract both innate and adaptive immune responses by modulating the function of several types of cells of the immune system, including monocytes (Mo), macrophages, dendritic cells (DCs), and T cells [4–8]. As a result of this, the expression profile of circulating pro- and anti-inflammatory cytokines and chemokines is altered, leading to chronic infection and persistent inflammation [4–8].

Until August 2011, the commercially available treatment options for HCV infection were limited to interferon-alpha- (IFN-*α*-) based therapies and ribavirin (RBV) for all genotypes [9–11]. IFN-*α* has antiviral activity and also enhances HCV-specific T cell responses; ribavirin, a nucleoside analogue, has a small direct activity against HCV but reduces hepatic inflammation [12]. It has been speculated that these two drugs act by modulating the immune system for HCV elimination [12].

In recent years, the introduction of several oral IFN-free direct-acting antiviral agents (DAAs) in clinical practice has proved to be a milestone in the management of HCV infection, increasing sustained virological response (SVR) rates up to 95-100% [13]. DAAs are molecules that target specific nonstructural proteins of the virus, with subsequent disruption of viral replication and spread to other cells. Since IFN-free DAA regimens specifically target various steps in the HCV life cycle, they can provide the opportunity to elucidate the relationship between HCV and the innate immune response, without the confounding effect of the IFN-*α*-induced immune modulation.

In the present study, we evaluated (i) the differences in DCs, Mo, and activated T cells in HCV-infected patients according to the stage of fibrosis and genotypes; (ii) the effect of IFN-based regimens and DAA-containing regimens with or without IFN on dynamic changes in circulating levels of Mo, DCs, and activated CD4 and CD8 cells; and (iii) the role of baseline values DCs, Mo, and activated T cells in predicting therapy response in patients on IFN-based regimens.

## 2. Materials and Methods

### 2.1. Study Population

The study was conducted in two outpatient clinics of a single referral center (Sapienza University, Rome). The study population included 73 patients with active HCV infection who were treated according to the current Italian national guidelines for HCV treatment [14]. Patients had no evidence of HIV or HBV infection or decompensated liver disease. Based on treatment response, patients were classified as follows: responders (R), if they achieved SVR, defined as undetectable HCV-RNA in blood 12 weeks after the end of therapy (SVR12). The subjects who stopped treatment because of virological failure or side effects were defined as non responders (NR).

During treatment and treatment follow-up, demographic, clinical, adverse events, and virological data were collected for all participants.

Data and blood samples were collected with respect to the patient's confidentiality and privacy.

### 2.2. HCV-RNA and HCV Genotype Testing

Plasma HCV-RNA levels were determined by RealTime PCR Roche Cobas TaqMan. HCV genotypes and subtypes 1a and 1b were determined by Abbott RealTime HCV Genotype II.

### 2.3. Liver Fibrosis Assessment

Liver stiffness (measured in kPa) was determined by transient elastography with the use of a FibroScan machine (Echosens). Advanced fibrosis (F4) was defined as a measure of liver stiffness (LSM) greater than or equal to 14.5 kPa; severe fibrosis (F3) was defined as a LSM value greater than or equal to 10; mild or no fibrosis (F0-F2) was defined as a LSM value less than 10 kPa [15].

### 2.4. Sample Handling

Venous blood samples were collected from each patient into EDTA-containing tubes (Becton±Dickinson Systems, San Jose, CA). Patients undergoing Peg-IFN-*α*/RBV treatment plus telaprevir (TVR) or boceprevir (BOC) had their peripheral blood samples collected at baseline (T0); after 1 (T1), 3 (T2), 6 (T3), and 12 (T end) months after the start of therapy; and within 4-6 months after the end (T post) or interruption of the therapy. Patients on IFN-free DAA regimens had peripheral blood samples collected at baseline (T0), 1 month after the start of T1, and 3 months after the end of the therapy (T post).

### 2.5. Enumeration of DCs and Monocytes

To enumerate DCs and monocytes, the same staining protocols previously described by our research group have been used [7, 16]. The analysis of the results was performed using FlowJo software (FlowJo 9.2, Tree Star, USA). Gating strategies are shown in additional [Supplementary-material supplementary-material-1]. Moreover, we stained the pDCs of 5 healthy donors in parallel with both the antibody panel used in our study (in which we identified pDC with CD123 and HLA-DR expression) and a new panel, which included BDCA-2, CD123, and HLA-DR, and we found that the two methods were equivalent.

### 2.6. CD4 and CD8 Activation Markers

To determine activated CD4 and CD8, the same staining protocols previously described by our research group have been used [16].

### 2.7. Statistical Analysis

All statistical analyses were performed using GraphPad Prism Software version 6.0 (Software MacKiev). Values are given as median and ranges (minimum and maximum values). The nonparametric Mann-Whitney test and the nonparametric Kruskal-Wallis ANOVA with Dunn's posttest were used to compare the differences in values. The nonparametric Wilcoxon matched paired test was applied to perform longitudinal analyses. All differences were considered statistically significant with *p* values less than 0.05.

## 3. Results

### 3.1. Study Population Characteristics

The study population included 73 patients; 25 were on INF-based treatment, including Peg-IFN-*α*/RBV plus first-generation protease inhibitors TVR and BOC; and 54 (of which 6 had previously failed with INF-based therapy) were treated with IFN-free DAA regimens with or without RBV. The demographic and clinical features of the patients are listed in Table 1.

In the IFN-based treatment group, 13 patients (52%) were responders and 12 (48%) were non responders, of which 33% NR for virological failure and 67% NR for side effects.

All the 54 patients treated with IFN-free DAA regimens were responders since they reached the SVR12; clinical features of patients are listed in Table 1. Among these patients, six had previously been treated with Peg-IFN-*α*+RBV and failed to achieve SVR and were retreated with an IFN-free DAA regimen after 1 year.

### 3.2. Immune System Parameters at Baseline

#### 3.2.1. DCs and Monocytes

Patients were stratified according to the stage of fibrosis.

**Figure 1:**
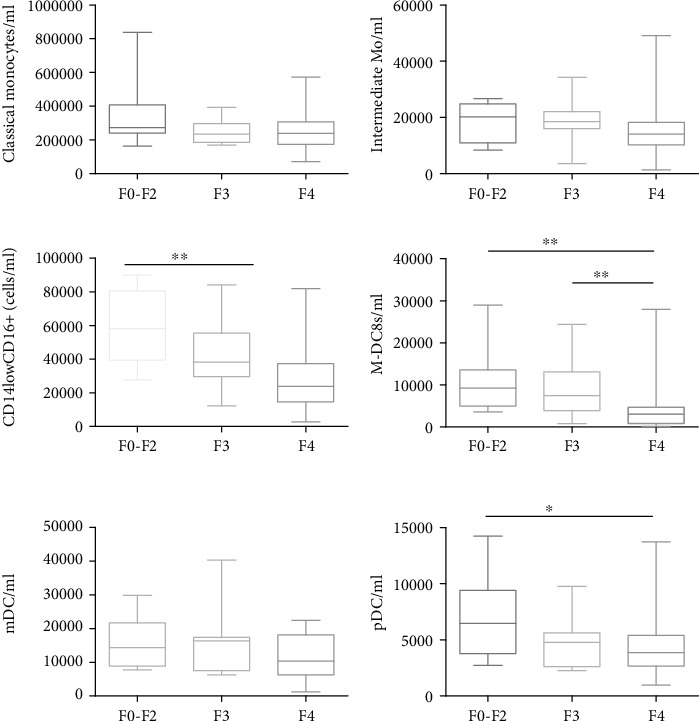
DC and monocyte count in 73 patients according to the fibrosis stage. 11 pts in the F0-F2 group, 16 in the F3 group, and 50 in the F4 group were enrolled. ^∗^*p* < 0.01, ^∗∗^*p* < 0.001, and ^∗∗∗^*p* < 0.0001.

An increase in nonclassical Mo in the F0-F2 group compared to the F4 one (median cells/ml (range): 58.106 cells/ml (27.889-89.971) vs. 23.856 cells/ml (2.783-82.076); *p* = 0.0016) was observed. Slan-DCs were drastically reduced in patients with F4 fibrosis compared to F3 and F0-F1 fibrosis (3.010 cells/ml for F4 (113.6-27.946), 7.441 cells/ml for F3 (3.919-13.121), and 9.258 cells/ml for F0-F1 (3578-28968), respectively; *p* = 0.0020). Also, a trend towards a decrease in plasmacytoid DCs (pDCs) from low fibrosis to high fibrosis was observed, with significant differences between F0-F2 and F4 (6.045 cells/ml (2.726-14.257) vs. 3.862 cells/ml (965.6-13746), respectively; *p* = 0.036). No significant differences were found in classical Mo, intermediate Mo, and myeloid DCs (mDCs) (Figure 1).

No significant differences were found in DCs and Mo counts in the different HCV genotypes (data not shown).

#### 3.2.2. CD4^+^ and CD8^+^ T Cell Activation

**Figure 2:**
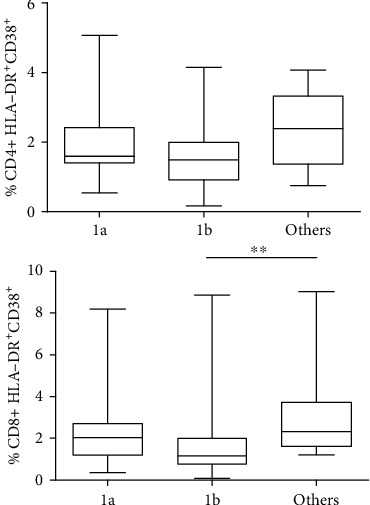
Activated CD4+ and CD8+ according to genotypes in all study population. 25 pts with genotype 1a, 39 pts with genotype 1b, and 15 pts with other genotypes were enrolled. ^∗^*p* < 0.01, ^∗∗^*p* < 0.001, and ^∗∗∗^*p* < 0.0001.

Regarding activated CD4^+^ and CD8^+^ T cells, we stratified patients according to the following: (i) the stage of fibrosis in which no differences were observed in activated T cells, for both CD4^+^ and CD8^+^ T cells (data not shown), and (ii) genotypes in which we observed a trend towards an increase in the percentage of CD4^+^ HLA-DR^+^CD38^+^ in patients with other genotypes compared to genotype 1b (2.39% (0.75-4.08) vs. 1.49% (0.17-2.39), respectively, *p* = 0.37). The median value (range) for genotype 1a was 1.59% (0.54-5.08). Regarding CD8^+^ T cells, we observed a significantly lower percentage of CD8^+^ HLA-DR^+^CD38^+^ T cells (1.03% (0-3.2)), compared to genotype 1a (2.85% (0.13-8.01)) and other genotypes (2.3% (1.2-8.9)) (*p* = 0.0059) (Figure 2). Considering all results, patients with genotype 1b showed the lowest levels of CD4^+^ and CD8^+^ T cell activation.

### 3.3. Dynamic Changes in Innate Immune Cells and T Cell Activation Markers during Treatment

#### 3.3.1. DCs and Monocytes

**Figure 3:**
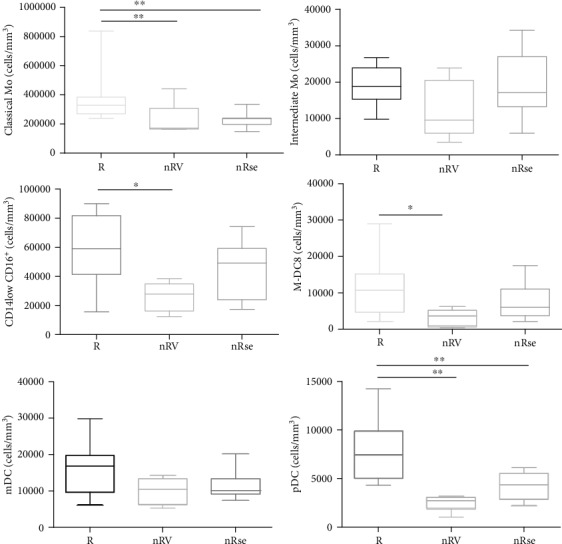
DCs and monocytes at baseline according to the response to IFN-based therapy: 13 responders (R), 4 non responders for virological failure (nRv), and 8 non responders for side effects (nRse). ^∗^*p* < 0.01, ^∗∗^*p* < 0.001, and ^∗∗∗^*p* < 0.0001.

*(1) INF-Based Therapy: Results*. Based on the outcome of treatment, we divided the patients into three groups: responders (R) and non responders (NR) for virological failure or for side effects (Figure 3).

At baseline, an increased value in classical Mo count in R compared to NR for virological failure and NR for side effects was found (median cells/ml (range): 327.622 cells/ml (239.355-838.879) for R vs. 171.934 cells/ml (161.766-441.222) for NR for virological failure vs. 235.550 cells/ml (146.885-333.473) for NR for side effects, respectively; *p* = 0.0046).

No differences were found in intermediate Mo count between R and NR for virological failure and for side effects (18.858 cells/ml (9.770-26.696) for R vs. 9.599 cells/ml (3.522-23.913) for NR for virological failure vs. 17.182 cells/ml (5.964-34.307) for NR for side effects, respectively; *p* = 0.056).

Nonclassical Mo were higher in R compared to NR for virological failure (58.958 cells/ml (15.620-89.971) vs. 27.889 cells/ml (12.326-38.397) respectively; *p* = 0.026), while no difference in their count was observed compared to NR for side effects (49.104 cells/ml (17.267-74.181)).

For what concerns slan-DCs, a higher value was found in R compared to NR for virological failure (10.764 cells/ml (2.102-28.968) vs. 3.578 cells/ml (398-6.248), *p* = 0.035); no difference was observed when comparing R with NR for side effects (6.078 cells/ml (2.102-17.494)).

No differences were found in the mDC count between the three groups. Median values and range were 16.870 cells/ml (6.305-29.876) for R, 10.508 cells/ml (5.282-14.370) for NR for virological failure, and 10.082 cells/ml (7.498-20.278) for NR for side effects, respectively.

A higher level of pDCs was found in R compared to NR for virological failure and NR for side effects (7.412 cells/ml (4.317-14.257) for R vs. 2.726 cells/ml (1.022-3.181) for NR for virological failure vs. 4.345 cells/ml (2.215-6.134) for NR for side effects, respectively; *p* = 0.0006).

Changes in innate immune system cell subsets from the baseline to the end of treatment were longitudinally analyzed in the three different groups, and the results are reported below.

**Figure 4:**
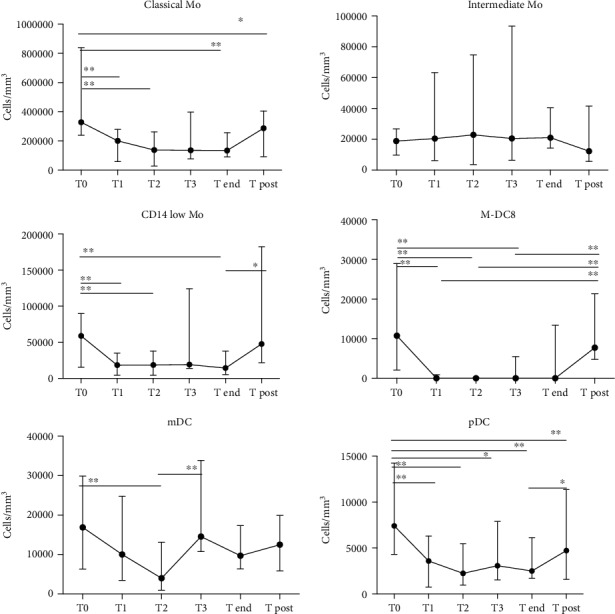
DC and monocyte dynamics during IFN-based treatment in the 13 responder patients. T0 = baseline; T1 = sample after 1 month of therapy; T2 = sample after 2 months of therapy; T3 = sample after 3 months of therapy; T end = sample after the end of therapy; T post = sample 3 months after the end of the therapy. ^∗^*p* < 0.01, ^∗∗^*p* < 0.001, and ^∗∗∗^*p* < 0.0001.

*(2) Responders*. In R, classical Mo progressively decreased during therapy at T1 month, T2 month, and T end and slightly increased at T post compared to T0 (baseline) (Figure 4). The median value at T0 was 327.622 cells/ml (239.355-838.879) compared to a value at T1 month of 200.419 cells/ml (28.911-279.513; *p* = 0.0005), at T2 month of 138.223 cells/ml (28.911-262.359; *p* = 0.001), at T end of 134.786 cells/ml (90.526-257.190; *p* = 0.0078), and at T post of 286.897 cells/ml (91.846-405.268; *p* = 0.04).

A slight increase in intermediate Mo was observed up to the end of treatment, and then, the values tended to drop at a slightly lower value than baseline, even though the difference was not statistically significant. The median values at the different time points were the following: T0 18.858 cells/ml (9.770-266.696), T1 month 20.476 cells/ml (6.248-63.332), T2 month 22.834 cells/ml (3.635-74.749), T3 month 20.562 cells/ml (6.418-93.379), T end 21.044 cells/ml (14.257-40.498), and T post 2.326 cells/ml (5.623-41.521).

Regarding nonclassical Mo, a statistically significant reduction during the course of treatment between T0 and T1 month (58.958 cells/ml (15.620-89.971) vs. 18.460 cells/ml (4.374-35.102); *p* = 0.0005), T0 and T2 month (58.958 cells/ml (15.620-89.971) vs. 18.517 cells/ml (4.601-38.113); *p* = 0.0005), and T0 and T end (58.958 cells/ml (15.620-89.971) vs. 14.342 cells/ml (5.453-37.942); *p* = 0.00078) was observed, whereas values at T post were significantly higher than those at the end of treatment (47712 cells/ml (21925-182498) vs. 14.342 cells/ml (5.453-37.942); *p* = 0.015).

Quite interestingly, slan-DCs were almost undetectable in peripheral blood samples after one month of treatment, with a subsequent trend towards recovery at the end of treatment and posttreatment (Figure 4). A significant difference was observed between T0 and T1 month to T3 month (10.764 cells/ml (2.102-28.968) for T0 vs. 0 cells/ml (0-908.8) for T1 month (*p* = 0.0005) vs. 0 cells/ml (0-170.4) for T2 month (*p* = 0.002) vs. 0 cells/ml (0-5510) for T3 month (*p* = 0.0039), respectively). Values at T post were significantly higher than those at T1 month to T3 month (7753 cells/ml (4771-21357) vs. 0 cells/ml (0-908.8) for T1 month (*p* = 0.002) vs. 0 cells/ml (0-170.4) for T2 month (*p* = 0.002) vs. 0 cells/ml (0-5510) for T3 month (*p* = 0.008)).

Considering mDCs, we observed a decrease up to the 12^th^ week of treatment, followed by an increase at week 24 and at the end of therapy. The median value at T0 was 16.870 cells/ml (6.305-29.877), at T1 month 10.025 cells/ml (3.408-24.765), at T2 month 3.976 cells/ml (965.6 to 13.121), at T3 month 14.541 cells/ml (10.735-33.853), at T end 9.684 cells/ml (6.362-17.324), and at T post 12.496 cells/ml (5.850-19.880).

Significant differences in mDC values were observed between T0 and T2 month (*p* = 0.0005) and between T2 month and T3 month (*p* = 0.00039).

The pDCs significantly decreased up to six months of therapy, with a subsequent progressive increase to values similar to those before the initiation of therapy.

There was a significant decrease in pDCs between T0 and T1 month (7.412 cells/ml (4.317-14.257) vs. 3.578 cells/ml (738.4-6.305), respectively; *p* = 0.0005), between T0 and T2 month (2.244 cells/ml (965.6–5.453), *p* = 0.0005), between T0 and T3 month (3.067 cells/ml (1.534-7.895), *p* = 0.0019), between T0 and T end (2.499 cells/ml (1.704-6.134), *p* = 0.0078), and between T0 and T post (4.714 cells/ml (1.590-11.360), *p* = 0.0098). Moreover, other significant differences were observed between T end and T post (*p* = 0.0031).

*(3) Non Responders for Virological Failure*. In NR for virological failure, no differences were observed neither in all Mo subsets nor in mDC and pDC counts during treatment (see additional [Supplementary-material supplementary-material-1]).

*(4) Non Responders for Side Effects*. In NR for side effects, no differences were observed in the monocyte subsets and mDC and pDC counts during the course of treatment (see additional [Supplementary-material supplementary-material-1]).

*(5) INF-Free Therapy: Results*. In all the patients who underwent DAA-based regimens, we observed a progressive decrease in classical Mo during therapy at T1 month and at T post compared to T0, with a statistically significant difference only between T0 and T post (*p* = 0.015). The median value at T0 was 284.568 cells/ml (142.738-573.055), at T1 month 184.578 cells/ml (29.659-325.634), and at T post 128.084 cells/ml (39.135-530.798) (see additional [Supplementary-material supplementary-material-1]).

#### 3.3.2. CD4 and CD8 Activation Markers

*(1) IFN-Based Therapy*. At the baseline, the percentage of CD4 and CD8 T cells did not differ between the three groups: R, NR for virological failure, and NR for side effects. For CD4 T cells, the median value for R was 32.3% (8-32.8), for NR for virological failure 30.1% (8.8-31), and for NR for side effects 33.4% (9-35.9) (data not shown). For CD8 T cells, the median value for R was 14% (7-32), for NR for virological failure 7% (4-22), for NR for side effects 8% (6-18.1) (data not shown).

The percentage of activated CD4 T cells, at baseline, did not differ between the three groups (R, NR for virological failure, and NR for side effects). The median value for R was 1.71% (0.36-8.28), for NR for virological failure 2.22% (0.17-3.66), and for NR for side effects 1.95% (1.4-4.42) (data not shown).

Similar results were found in CD8^+^-activated cells, for which the median value for R was 1.73% (0.19-8.64), for NR for virological failure was 2.2% (0.08-11.11), and for NR for side effects was 1.59% (0.8-8.2) (data not shown).

*(2) Responders*. In R patients, the percentage of CD4 and CD8 T cells did not change during the therapy. The median value for CD4 T cells at T0 was 32.3% (8-32.8), at T1 month 30.3% (7-32), at T2 month 30.5% (7-31), at T3 month 31% (8-32), at T end 31.2% (8.5-33), and at T post 31.1% (8.1-31). Regarding CD8 T cells, the median value at T0 was 14% (7-32), at T1 month 15% (7.1-33), at T2 month 15.1% (7-31), at T3 month 14.8% (8-31), at T end 14.5% (8.5-31) and 13.9% (7.2-31), and at T post 14% (7.5-32).

**Figure 5:**
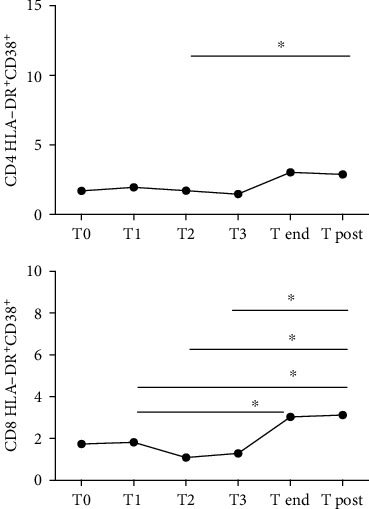
Activated CD4 HLA-DR^+^CD38^+^ and CD8 HLA-DR^+^CD38^+^ dynamics in 25 patients under IFN-based treatment with sustained virological response. T0 = baseline; T1 = sample after 1 month of therapy; T2 = sample after 2 months of therapy; T3 = sample after 3 months of therapy; T end = sample after the end of therapy; T post = sample 3 months after the end of the therapy. ^∗^*p* < 0.01, ^∗∗^*p* < 0.001, and ^∗∗∗^*p* < 0.0001.

In R patients, we observed an overall increase in CD4^+^- and CD8^+^-activated cells during the course of therapy, which seemed to persist even after the end of the treatment (Figure 5). The percentage of CD4^+^ HLA-DR^+^CD38^+^ cells increased significantly between T2 month and the end of the therapy (*p* = 0.046), and additionally, we observed an increasing trend between T1 month and T end, between T2 month and T end, and between T3 month and T end. The median value at T0 was 1.65% (0.36-3.05), at T1 month 2.07% (1.52-3.98), at T2 month 2.44% (0.51-5.63), at T3 month 1.47% (1.15-3.21), at T end 3.28% (2.26-5.24), and at T post 2.85% (1.19-7.26).

The percentage of activated CD8^+^ T cells remained substantially stable during therapy up to T3 month, with the following significant increase between T3 month and T post (*p* = 0.015). Moreover, the percentage of activated CD8^+^ cells at T post was higher compared to that at T1 month (*p* = 0.046) and T2 month (*p* = 0.046). The median value at T0 was 1.25% (0.19-8.64), at T1 month 1.91% (0.7-6.27), at T2 month 1.35% (0.37-5.28), at T3 month 1.29% (0.57-4.8), at T end 3.28% (2.26-5.24), and at T post 3.91% (1.16-8.19).

*(3) Non Responders for Virological Failure*. In NR patients for virological failure, the percentage of CD4 and CD8 T cells did not change during the therapy. The median value at T0 was 30.1% (8.8-31), at T1 month 29.5% (9-30), at T2 month 30% (8.3-29.8), at T3 month 30.2% (8-30), at T end 31.4% (8.7-32), and at T post 31.2% (8.5-31.5). Regarding CD8 T cells, the median value at T0 was 7% (4-22), at T1 month 7.5% (5-23), at T2 month 7.1% (4-22), at T3 month 7.3% (5.3-19), at T end 7.8% (4.5-2) and 13.9% (6.9-22), and at T post 7.3% (5-22.3).

No differences were observed in the levels of CD4^+^ and CD8^+^ cell activation during the course of therapy. For CD4^+^ HLA-DR^+^CD38^+^ cells, the median value at T0 was 2.43% (0.17-3.66), at T1 month 1% (0.89-1.13), at T2 month 1.03% (0-95-1.11), at T3 month 0.57% (0.57-0.57), at T end 1.57% (1.41-2.76), and at T post 1.57% (1.28-2.74).

For CD8^+^ HLA-DR^+^CD38^+^ cells, the median value at T0 was 2.82% (0.08-11.11), at T1 month 0.43% (0.27-0.68), at T2 month 0.6% (0.18-1.03), at T3 month 0.75% (0.75-0.75), at T end 3.46% (2.82-4.11), and at T post 1.71% (0.83-3.63) (data not shown).

*(4) Non Responders for Side Effects*. In NR patients for side effects, the percentage of CD4 and CD8 T cells did not change during the therapy. The median value for CD4 T cells at T0 was 33.4% (9-35.9), at T1 month 32.3% (9.3-34), at T2 month 32.5% (9.1-34.5), at T3 month 32% (9.3-34), at T end 33.2% (8.5-34), and 33% (9.1-35.2) at T post.

Regarding CD8 T cells, the median value at T0 was 8% (6-18.1), at T1 month 8.2% (6.3-17.6), at T2 month 7.9% (5.9-17.4), at T3 month 8.1% (6-18), at T end 7.9% (6.5-19) and 7.9% (6.9-18), and at T post 7.9% (6.2-19).

A significant difference in the percentage of CD4^+^ HLA-DR^+^CD38^+^ cells was observed between T0 and T post (1.95% (1.4-4.42) vs. 2.62% (1.68-6.52), *p* = 0.015). The median percentage at T1 month was 0.69% (0.56-1.64), at T2 2.07% (1.14-3.65), and at T3 month 2.39% (2.39-2.64).

No differences in the percentage of CD8^+^ HLA-DR^+^CD38^+^ cells were found. The median value at T0 was 1.59% (0.8-8.2), at T1 month 1.72% (0.52-2.59), at T2 month 5.4% (2.3-8.78), at T3 month 9.04% (9.04-10.04), and at T post 2.05% (1.29-8.94) (data not shown).

*(5) INF-Free Therapy*. During the therapy, the percentage of CD4 and CD8 T cells did not change (data not shown). For CD4 T cells, the median value was 30.6% (5-53.5) while for CD8 T cells, the median value was 15.1% (2-25). During therapy, no changes in CD4 and CD8 T cells were observed. For CD4 T cells, the median value at T0 was 30.6% (5-53.5), at T1 month 30.1% (5.2-52), and at T post 30.2% (5.6-53). For CD8 T cells, the median value at T0 was 15.1% (2-25), at T1 month 15.5% (2.4-24), and at T post 15.2% (2.3-24.5).

The percentage of CD4^+^ HLA-DR^+^CD38^+^ cells during treatment remained almost constant, with a slight decrease at T post (1.31% (0.13-4.9)) compared to T0 (1.59% (0.5-6.5)) (*p* = 0.045). The median value at T1 month was 1.36% (0.98-2.93).

**Figure 6:**
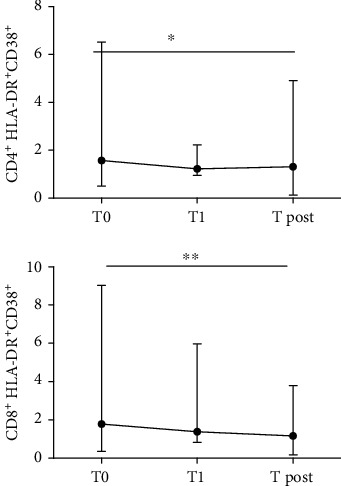
Activated CD4^+^ HLA-DR^+^CD38^+^ and CD8^+^ HLA-DR^+^CD38^+^ dynamics in 54 patients under IFN-free treatment. T0 = baseline; T1 = sample after 1 month of therapy; T post = sample after 3 months the end of the therapy. ^∗^*p* < 0.01, ^∗∗^*p* < 0.001, and ^∗∗∗^*p* < 0.0001.

A greater decrease between T0 1.72% (0.36-9.02) and T post 1.19% (0.17-4.18) was observed in CD8^+^-activated cells (*p* = 0.0038). The median value at T1 month was 1.4% (0.83-8.59) (Figure 6).

## 4. Discussion

The last few years have seen extraordinary advances in the management of patients with chronic HCV infection.

The use of a triple therapy which combined first-generation DAAs with pegylated interferon (Peg-IFN) and ribavirin (RBV) significantly improved the chances of achieving sustained virological response (SVR) rates to over 60%. However, treatment with telaprevir and boceprevir was intended for genotype 1 HCV-infected patients and antiviral potency was counteracted by considerable side effects, in particular an increased number of bacterial infections [17, 18].

In our study, patients undergoing IFN-based therapy showed a successful rate of 67% in accordance with what is known from the literature [19, 20].

In almost half of nonresponder patients, the interruption of therapy was mainly due to neutropenia and infectious diseases (sepsis and pneumonia), in line with previous reports of the association with an increase in treatment-related adverse events compared to the former standard therapy without protease inhibitors. The mechanism of this increased susceptibility to infections seems to be related to the in vitro inhibition of neutrophil elastase activity [21, 22].

IFN-free treatments consisting of combinations of second-generation DAAs, with or without ribavirin, showed impressive SVR rates and a safer profile. In our cohort, the success rate was 100% and no patients interrupted the therapy for virological failure or for adverse effects.

In the present study, we evaluated the differences in DCs, Mo, and activated T cells in all HCV-infected patients according to fibrosis stage and genotype.

The innate immune system plays a pivotal role in the host-virus interactions during the entire natural course of the disease, and IFNs are the central cytokines responsible for the induction of an antiviral state in cells and for the activation and regulation of the cellular components of innate immunity [23]. IFN-*α* has potent antiviral activity, and it acts by inducing IFN-stimulated genes (ISGs), which establish a non-virus-specific antiviral state within the cell [24]. Exogenously supplied recombinant IFN-*α* binds to and activates cellular receptors, leading to the same response as with the endogenous one.

Dendritic cells (DCs) are professional antigen-presenting cells (APCs), playing a key role in the innate immune system in orchestrating the quality and potency of downstream adaptive immune response, through the uptaking and processing of viral antigens, as well as by releasing cytokines to efficiently prime both CD4^+^ helper T cells and CD8^+^ cytotoxic T lymphocytes (CTLs) [25].

Two major subsets of DCs can be readily purified from human peripheral blood: plasmacytoid (p)DCs and conventional or myeloid (m)DCs [25]. pDCs and mDCs differ markedly in their ability to capture, process, and present antigens; express costimulatory molecules; and produce cytokines [26].

Slan-DCs are a third subset of DCs that were identified in peripheral blood using the monoclonal antibody M-DC8, which binds to 6-sulfo LacNac (slan), a carbohydrate moiety of the P selectin glycoprotein ligand 1 (PSGL-1) [27]. However, on a two-dimensional flow cytometry dot plot of CD14 and CD16 expression in peripheral blood mononuclear cells (PBMCs), slan-DCs in part overlap with CD14^dim^CD16^+^ monocytes [28], suggesting that they might actually represent a subset of nonclassical monocytes [29].

Regarding their function, blood slan-DCs have been described as potent proinflammatory cells, based on their ability to produce large amounts of tumor necrosis factor-alpha (TNF-*α*) and IL-12p70 upon stimulation with toll-like receptor (TLR) ligands [28].

In our study, all HCV-infected patients were analyzed in relation to the stage of fibrosis. Interestingly, an increase in nonclassical Mo, slan-DCs, and pDCs in peripheral blood was found in patients with low fibrosis compared with those with high fibrosis. This could reflect a higher level of these cells in peripheral blood during the early stages of fibrosis, followed by a reduction due to their migration to the liver when fibrosis is progressing. Indeed, peripheral blood monocytes constantly enter the liver to replenish hepatic macrophages, and the number of infiltrating monocytes increases during liver inflammation and modulates fibrogenesis [30]. In fact, during fibrogenesis, there is an infiltration of leukocytes and monocytes/macrophages into the liver; this infiltration is the key for the activation of hepatic stellate cells and subsequent fibrosis; moreover, reducing liver-infiltrating macrophages, it is possible to attenuate fibrosis in some models [30]. During HCV infection, there is a continuous recruitment of monocytes and macrophages into the liver where under local signals became able to secrete a variety of proinflammatory, profibrotic, and anti-inflammatory cytokines and growth factors [29].

Monocytes are attracted to the site of infection and are exposed to viral RNA and protein, which can lead to their activation [31, 32]. Nonclassical monocytes (CD14^++^CD16^+^) preferentially accumulate in the chronically inflamed human liver as a consequence of enhanced recruitment from blood and local differentiation from classical CD14^++^CD16^−^ monocytes [29]. Nonclassical monocytes are major modulators of fibrogenesis in the liver producing profibrogenic cytokines and chemokines. Mascia et al. recently [8] described how monocyte-derived cytokines such as CXCL-10 and sCD14 are increased in all stages of fibrosis, underlying the presence of immune activation from F0 to F4 [8, 33]. Slan-DCs seem to contribute to the pathogenesis of chronic inflammatory diseases such as Crohn's disease, rheumatoid arthritis, psoriasis, and HIV since they infiltrate the inflamed ileal mucosa, skin, and synovial tissue [16, 28, 34]. They are a major source of tumor necrosis factor-*α*, a pleiotropic cytokine produced even by macrophages/monocytes in response to bacterial LPS, and are significantly expanded in patients with bacterial sepsis [34]. TNF-*α* has been implicated in the pathogenesis of chronic liver inflammation-activating resident HSCs into fibrogenic myofibroblasts. However, there are currently no data on the possible migration of slan-DCs into the inflamed liver during the progress of fibrosis. The subset of plasmacytoid DCs (pDCs) is considered to be the front line in antiviral immunity, owing to the rapid production of high amounts of type I interferon in response to viruses [35]. Patients with chronic HCV infection have a reduced ability to produce INF after in vitro stimulation of pDCs due to a decrease in their absolute number, an impairment in their function, and an increase in their homing to the liver [32].

Various studies have shown that CD4^+^ helper T cell- and CD8^+^ cytotoxic T cell-mediated immune responses determine the outcome of HCV infection. Thus, spontaneous viral clearance of HCV infection is characterized by vigorous and sustained specific CD4^+^ and CD8^+^ T cell responses during the acute phase of infection, while in contrast chronic infection is correlated with late, transient, weak, or narrowly focused CD4^+^ and CD8^+^ T cell responses [36, 37].

We focused our analysis on activated CD4^+^ and CD8^+^ cells, and interestingly no differences in activated CD4^+^ and CD8^+^ were found in HCV-infected patients stratified for fibrosis stage. Regarding different genotypes, a lower activation of CD8^+^ cells was observed in patients with genotype 1b compared to other genotypes. Genotype 1b has historically been associated with a lower likelihood of selection of resistant variants and a better response to IFN-based therapy, compared to other genotypes [38]. In this setting, the lower impact on the activation of peripheral CD8^+^ cells can have an impact on the response to treatment.

To investigate the effect of IFN-based regimens and DAA-containing regimens with or without IFN on dynamic changes in circulating levels of monocytes (Mo) and DCs and activated CD4 and CD8 cells, we performed a longitudinal study.

During the course of INF-based regimens, we observed a decrease in pDCs after 1 month of therapy, which persisted up to the end of therapy, with subsequent increase after the interruption of treatment, although without a restoration to pretreatment values. mDCs tended to remain stable over the treatment course, although a decrease in number was observed at month 3, followed by a recovery at month 6.

No differences in both pDCs and mDCs were found in patients who failed due to virological failure or side effects.

Regarding monocytes, a decrease in classical monocytes, nonclassical monocytes, and slan-DCs was observed after 1 month of therapy that persisted after 6 months. No differences were observed in non responders.

These variations in the levels of circulating innate immune cells could be a consequence of the IFN-induced depression of bone marrow activity, as suggested by the well-known reduction in granulocytes during treatment [39], and could explain the increased risk of bacterial infections observed in our study population.

On the other hand, DAA therapy lacks the immunomodulatory effects of IFN, possibly explaining the absence of differences in DCs and Mo counts during interferon-free treatments.

To address the dynamics of immune activation in HCV infection, we analyzed CD38 and HLA-DR expression on the CD4^+^ and CD8^+^ T cells during the two different treatments.

Both responders and non responders for side effects had increased levels of activation of CD4^+^ cells over the course of IFN-based therapy, which remained persistently elevated even after the end of treatment in non responders for side effects, while no alterations were observed in non responders for virological failure. On the other hand, the percentage of activated CD8+ cells was significantly increased only in responders during the course of IFN-based regimens.

Our results are in contrast with previously published data on the decrease in immune activation in the T cell compartment after IFN-based therapy [40]. However, Radkowski et al. reported increased levels of immune activation associated with persistence of HCV-RNA in PBMCs after successful treatment of chronic HCV infection, which could be linked to an increased risk of developing immune-mediated extrahepatic complications of HCV infection in some patients [41].

On the contrary, during the course of DAA-based regimens, we observed a decrease in activated CD8^+^ T cells after the end of therapy. This highlights the importance of immune activation in the pathogenesis of chronic HCV infection, considering the reduced immune activation following complete viral clearance as a consequence of successful treatment with DAAs.

Finally, the observation of a higher number of classical and nonclassical monocytes, together with increased levels of pDCs and slan-DCs in responders, could suggest a possible role of the evaluation of these cell subsets in predicting treatment response during the course of IFN-based regimens. In fact, those cell subsets are involved in the immune response to HCV and have been found in peripheral blood and in the liver of patients with chronic HCV, suggesting a primary role in the immune response to the virus [31, 34, 35]. Thus, higher levels of these subpopulations of cells of the innate immune system might enhance the response against the virus and facilitate its eradication.

There are several limitations in our study. Firstly, we did not evaluate the functional status of circulating monocytes and DCs, since the analysis was limited to numerical changes in peripheral blood samples. Moreover, HCV-infected patients were not treated with the same DAA regimen, and this may have had an impact on the results, due to the possibility of differential effects of the various DAAs on immune function.

Taken together, these results could suggest that increased circulating levels of specific cell subsets within the innate immune compartment could promote antiviral response during the course of IFN-based regimens and could help in predicting the outcome of treatment in some patients.

## Figures and Tables

**Table 1:** Characteristics of study population.

	INF-free therapy *N* 54	INF-based therapy *N* 25
Age, median (range)	61 (40-78)	52 (25-70)
Sex male, *n* (%)	43 (80%)	20 (80%)
Genotypes, *n* (%)		
1a	17 (31%)	8 (32%)
1b	22 (41%)	17 (68%)
Others	15 (28%)	
FIB-4, median (range)	2.91 (0.9-11.84)	2.08 (0.5-20.44)
FibroScan (kPa), median (range)	22.7 (10.6-248)	11.6 (1.3-37.4)
HCV-RNA (∗10^6^ cp/ml), median (range)	0.88(0.0014-3.12)	2.28 (0.78-7.95)
ALT (UI/l), median (range)	91 (28-407)	105 (42-288)
AST (UI/l), median (range)	69 (22-247)	73 (26-280)
PLT (∗10^9^/l), median (range)	139 (28-340)	170 (34-290)
Cirrhotic, *n* (%)	40 (70%)	9 (36%)
Fibrosis stage		
F0-F2, *n* (%)	1 (2%)	10 (40%)
F3, *n* (%)	10 (19%)	6 (24%)
F4, *n* (%)	41 (79%)	9 (36%)

## Data Availability

All data used to support the findings of this study are included within the article and in the additional materials.

## Supplementary Materials

Supplementary 1 Additional File 1: gating strategy for DCs and monocytes.

Supplementary 2 Additional File 2: DC and monocyte count in virologically nonresponder patients during IFN-based treatment.

Supplementary 3 Additional File 3: DC and monocyte count in nonresponder patients for side effects during IFN-based treatment.

Supplementary 4 Additional File 4: DC and monocyte count in patients under IFN-free treatment.
