# Supplementary figures and images for: Impact of IFN-Free and IFN-Based Treatment on Blood Myeloid Dendritic Cell, Monocyte, Slan-DC, and Activated T Lymphocyte Dynamics during HCV Infection
Source: J Immunol Res. 2020 Mar 16;2020:2781350. doi: 10.1155/2020/2781350 (PMC7102477; doi:10.1155/2020/2781350)

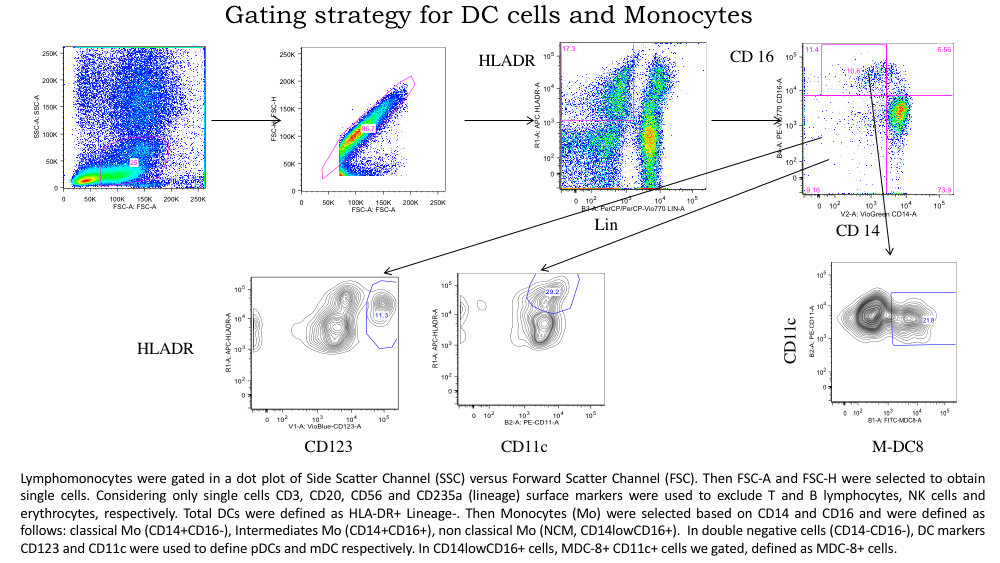

Supplement: Supplementary 1 — Additional File 1: gating strategy for DCs and monocytes. [file 2781350.f1.tiff]

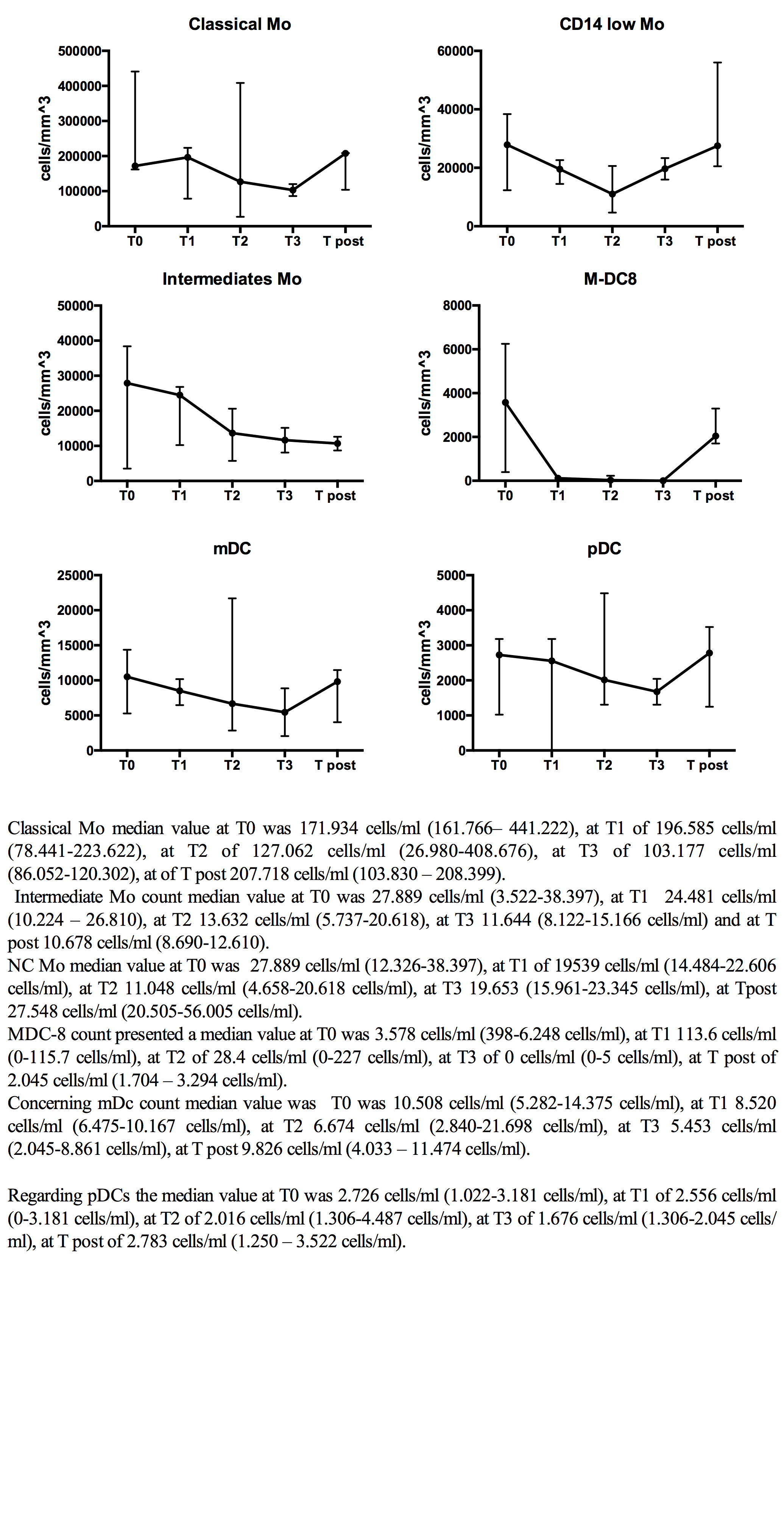

Supplement: Supplementary 2 — Additional File 2: DC and monocyte count in virologically nonresponder patients during IFN-based treatment. [file 2781350.f2.tiff]

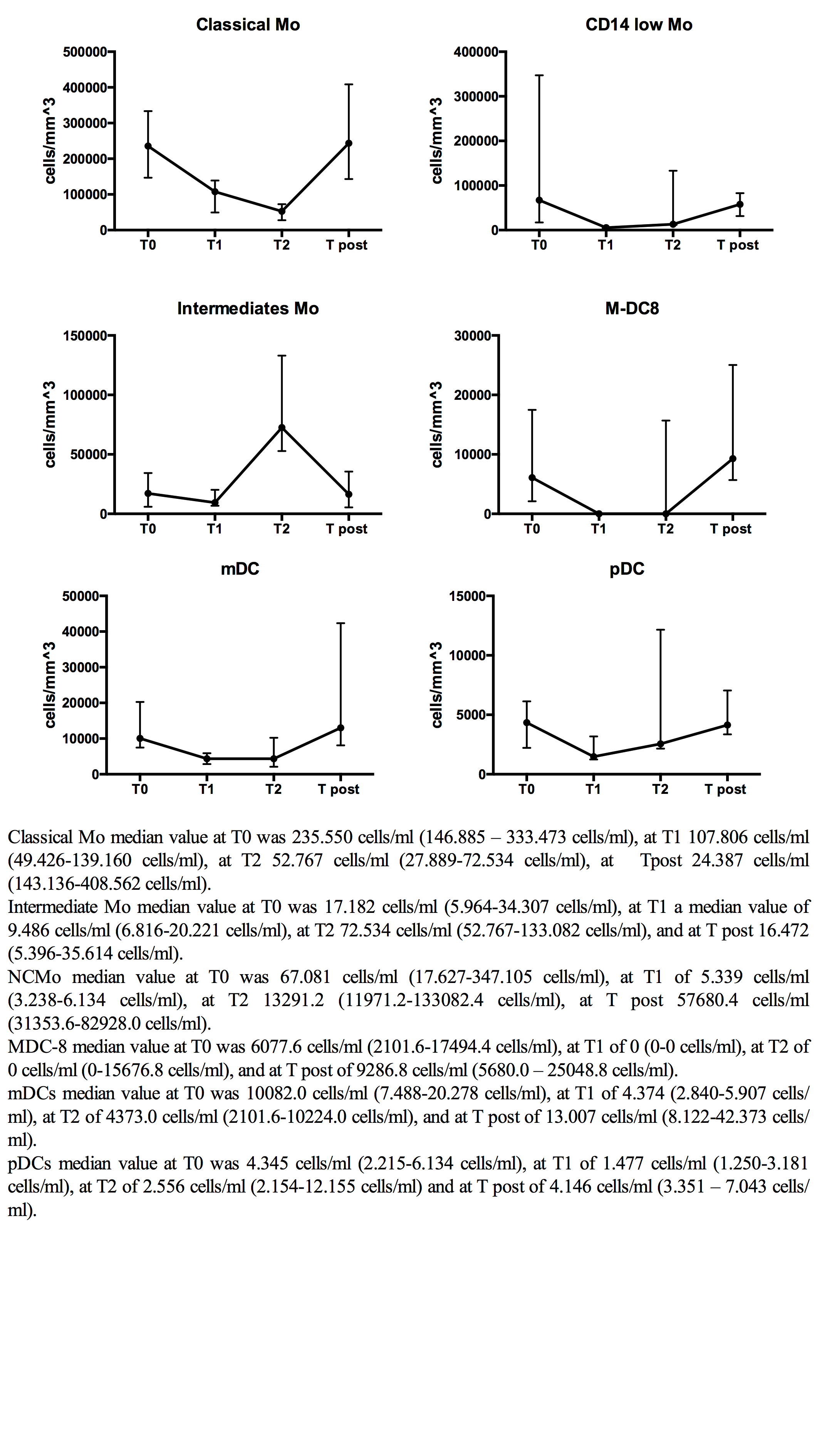

Supplement: Supplementary 3 — Additional File 3: DC and monocyte count in nonresponder patients for side effects during IFN-based treatment. [file 2781350.f3.tiff]

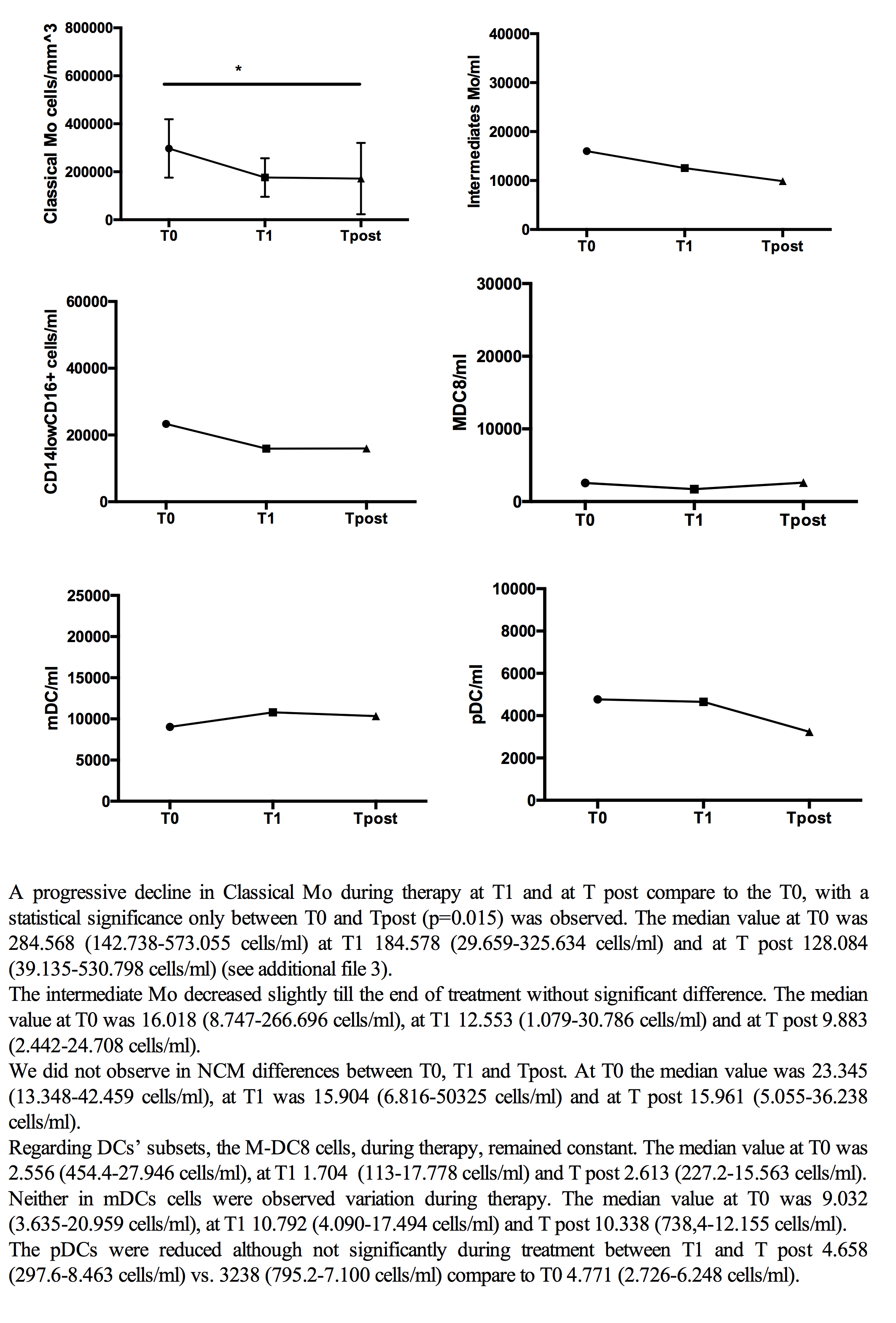

Supplement: Supplementary 4 — Additional File 4: DC and monocyte count in patients under IFN-free treatment. [file 2781350.f4.tiff]
